# Coexistence of Write Once Read Many Memory and Memristor in blend of Poly(3,4-ethylenedioxythiophene): polystyrene sulfonate and Polyvinyl Alcohol

**DOI:** 10.1038/srep38816

**Published:** 2016-12-12

**Authors:** Viet Cuong Nguyen, Pooi See Lee

**Affiliations:** 1 School of Materials Science and Engineering, Nanyang Technological University, 50 Nanyang Avenue, Singapore 639798, Singapore

## Abstract

In this work, the coexistence of Write Once Read Many Memory (WORM) and memristor can be achieved in a single device of Poly(3,4-ethylenedioxythiophene): polystyrene sulfonate (PEDOT: PSS) and Polyvinyl Alcohol (PVA) blend organic memory system. In memristor mode, the bistable resistance states of the device can be cycled for more than 1000 times. Once a large negative bias of −8V was applied to the device, it was switched to permanent high resistance state that cannot be restored back to lower resistance states. The mechanism of the memristor effect can be attributed to the charge trapping behaviour in PVA while the WORM effect can be explained as the electrochemical characteristic of PEDOT: PSS which harnesses the percolative conduction pathways. The results may facilitate multipurpose memory device with active tunability.

Organic materials have been paid much attention in soft electronic devices such as transistor or memory due to its inherently flexible and cost-effective processes. Utilizing organic materials for flexible and novel applications has been an interesting topic for both industrial purpose as well as basic research^1^,^2^. Recent concept of two terminal resistive switching device or memristor has open up another exciting research field utilizing organic materials. In polymer and composites, there have been many interesting reports about resistive switching phenomena such as Dynamic Random Access Memory (DRAM)^3^, Static Random Access Memory (SRAM)^4^, Write Once Read Many Memory (WORM)^5^ and Rewritable memory (FLASH)^6^. The mechanisms are often complex and Density Functional Theory (DFT) simulations have been employed to get better understanding. There are many mechanisms that have been suggested for resistive switching phenomena in organic materials namely, donor-acceptor charge transfer complex^3^, charge trapping^7^ and modulation of dopant in conjugated polymer^8^ or electrode metal migration^9^. The large variety of resistive switching phenomena in different organic materials point to an interesting question of whether one can obtain two (or more) resistive switching modes or mechanisms in a single device through active manipulating voltage bias and therefore realizing a reconfigurable switching device concepts and dynamics to reproduce relevant computational primitives that potentially emulate biophysics of real synapses and neurons.

Alongside with research on switching characteristics of organic materials, recent works have also concentrated on water soluble organic materials resistive memory for biocompatible and environmental friendly applications^10^. Some natural macromolecules such as protein, DNA and silk have been utilized for biocompatible and biodegradable resistive switching memory^11^. Sun *et al*. have used water soluble Polyurethane and organic semiconductor blends for various resistive memory applications such as Write Once Read Many Memory or rewritable (Flash) memory^12^. In this work, we demonstrate the coexistence of Write Once Read Many Memory and memristor in a single device using the blend of Poly(3,4-ethylenedioxythiophene)-poly(styrenesulfonate) (PEDOT: PSS) and Poly Vinyl Alcohol (PVA). Both materials are biocompatible and water soluble/ dispersible. Although PEDOT: PSS has been utilized in resistive memory device, reports on electrical memory behaviour associated with blends of PEDOT: PSS and PVA is scanty^13^. Furthermore, report on the coexistence of WORM memory and memristor in a single device is also sparse in organic resistive memory device literature^14^. We envisage that the future possibility of fabricating polymer based resistive switching single device that can exhibit various modes of switching using electrical stimuli as well as optical or magnetic stimuli, may intrigue research into using organic solids for multipurpose memory devices with active tuning stimuli^15^,^16^.

## Results and Discussion

**Figure 1:**
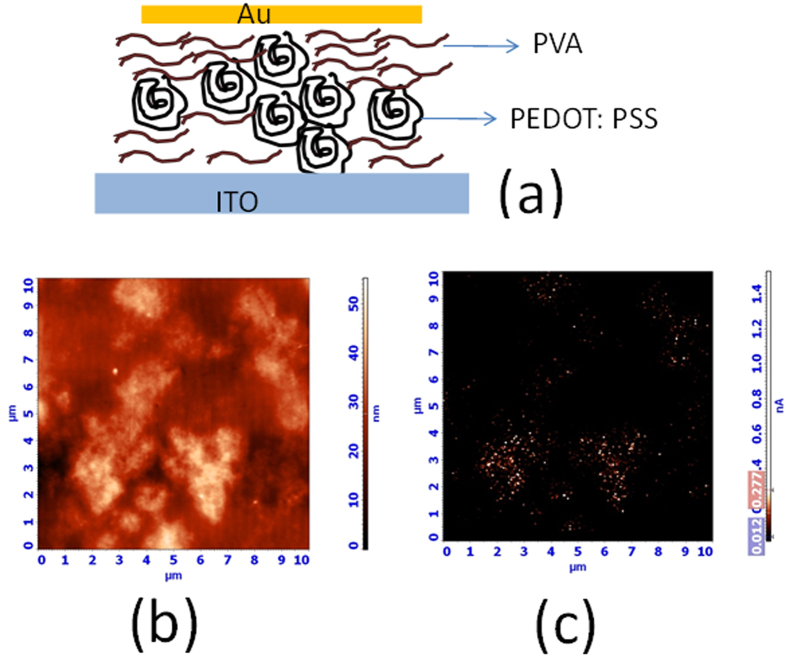
(**a**) Device structure of the blend PEDOT: PSS - PVA, (**b**) and (**c**) AFM image and corresponding cAFM image of the blend respectively.

The structure of the memory device is shown in Fig. 1a. In Fig. 1b and c, the Atomic Force Microscope (AFM) and conducting Atomic Force Microscope (cAFM) images of the films are shown. The AFM image shows aggregations of domains. The corresponding cAFM (at 0.2 V tip biased) at the same location shows that those aggregations can conduct electrical current. It is therefore reasonable to assign those aggregations as PEDOT: PSS rich domains where charges can transport through conjugated bonds of conducting polymer PEDOT: PSS. Non-conducting area is identified as the PVA- rich matrix where the relative insulative nature of PVA hinders electrical transport.

**Figure 2:**
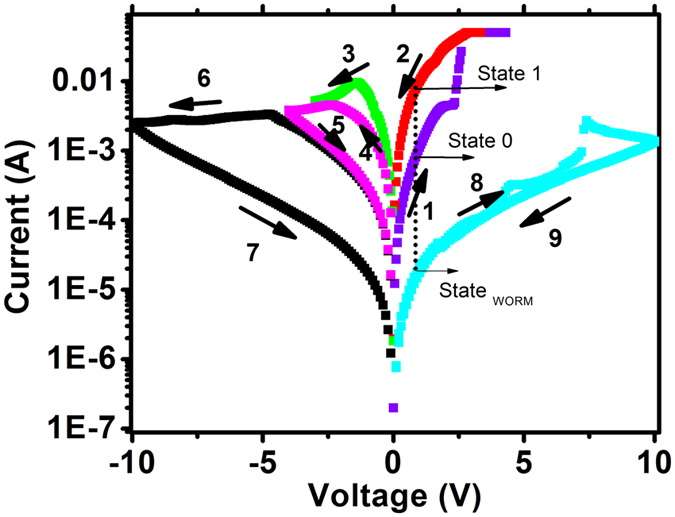
Coexistence of memristor and WORM in PEDOT: PSS – PVA blend device. When voltage was cycled between −3 V and 4 V, memristor is unraveled. After sweeping toward −8 V (arrow 6, 7), the device’s state is switched to WORM memory (state _WORM_) and unable to be restored back to state 0 or 1 (arrow 8, 9).

**Figure 3:**
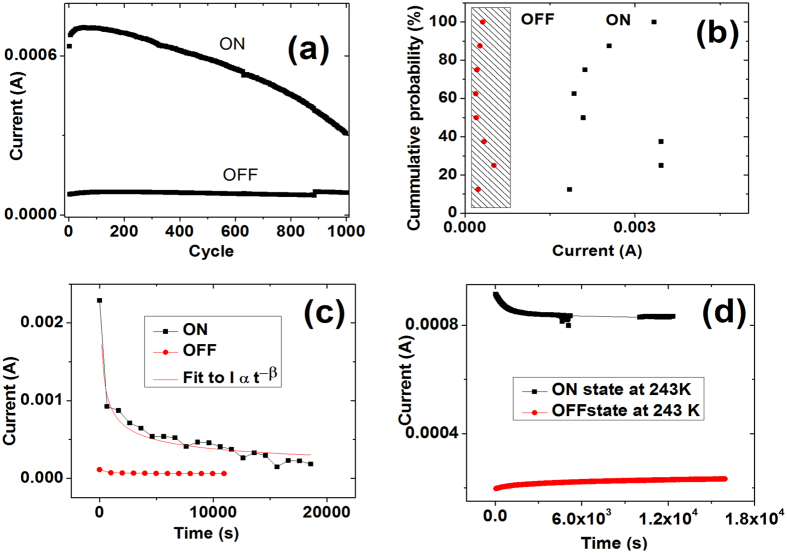
(**a**) Cycling and (**b**) cumulative probability of 8 random devices from 3 different batches of PEDOT: PSS – PVA blend devices. Current was extracted at 0.5 V. (**c**) and (**d**) retention of device states at 243 K and ambient condition respectively.

The current voltage (I–V) characteristic of the device for blend ratio 1: 10 is shown in Fig. 2. The reversible memristor with self-crossing characteristic can be achieved in voltage range of −3 V to 4 V with state 1 and state 0 denoted as the On state and Off state^17^. This memristor effect can also be obtained in N_2_, O_2_ rich environment and vacuum environment as shown in Fig. S1. The original state of the device is in state 0. After applying threshold positive voltage of 4 V, the state 0 was abruptly switched to state 1. The state 1 can be switched back to state 0 by sweeping bias to −3 V. A memory window with on/off ratio of around 10 can be reached with a read voltage at 0.3 V. The device resistance states can be cycled for more than 1000 times by writing at 4 V, erasing at −3 V and reading at 0.3 V as shown in Fig. 3a. In Fig. 3b, the replication of the memristor characteristic of 8 devices randomly chosen from 3 different batches is shown; this demonstrates the consistency of the observed phenomenon. As shown in Fig. 2, after voltage is swept from 0 to −10 V, the resistance of the device stays at high resistance state (denoted as state _WORM_) and cannot be restored back to state 1 or state 0 by opposite voltage sweep from 0 V to 10 V. A Write Once Read Many (WORM) memory characteristic was unveiled after this negative sweep where state _WORM_ cannot be restored back to state 1 or state 0. Hence, in a single device, through the control of voltage bias, one can obtain both memristor and WORM memory behavior. The retention characteristic of state 1 and state 0 are shown in Fig. 3c and d. In the device tested in air, very fast decay was recorded; nonetheless 10^4^ s retention can be obtained (Fig. 3c). Much better retention time can be obtained after testing the device in vacuum and low temperature 243 K (Fig. 3d). State 1 decay is much reduced when tested at low temperature.

**Figure 4:**
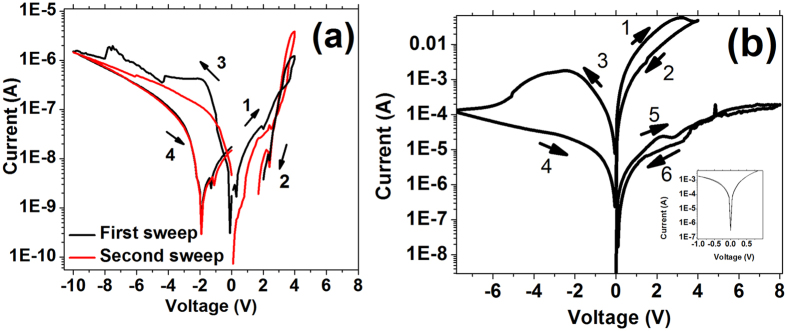
(**a**) I–V characteristic of Au/PVA/ ITO (**b**) I–V characteristic of Au/ PEDOT: PSS/ ITO. Inset of (**b**) shows Ohmic shape I–V at small voltage (−1 V to 1 V) of pristine Au/ PEDOT: PSS/ ITO device.

The I–V behavior of the pristine PVA and PEDOT: PSS control samples are shown in Fig. 4a and b respectively. As clearly seen in Fig. 4a, the current level of PVA film is lower and therefore more insulative compared to the PEDOT: PSS – PVA blend film; the leakage current can stem from ionic defects or ions trapped inside PVA film. However, no threshold set voltage or memory switching was found in the pristine PVA film. We can therefore rule out the possibility of metallic filaments formation in the resistive switching mechanism of this work. While applying higher voltage (up to −10 V) to the PVA control device, one can confirm the absence of WORM characteristics. The I–V of PEDOT: PSS film shows the WORM memory characteristic without the trace of memristor characteristic. Hence, it is reasonable to attribute the memristor effect of the PEDOT: PSS - PVA blended device to the polymer blend itself rather than any of its individual material component; while the WORM effect can be attributed to the PEDOT: PSS. WORM memory in PEDOT: PSS has been explained extensively as redox reactions which dedope conducting PEDOT^+^ to non-conducting PEDOT^0^ 18. Furthermore, it has also been pointed out that electrochemical characteristic (including migration of PSS chains) of PEDOT: PSS also distorted the top electrode contact and PEDOT: PSS conduction pathways which resulted in suppression of electrical transport and hence gave rise to a WORM memory phenomenon^19^,^20^.

**Figure 5:**
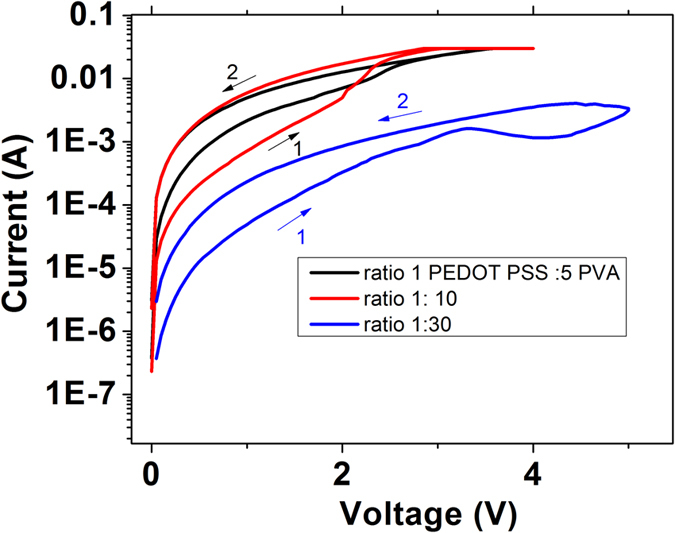
I–V of Au/blend PEDOT: PSS and PVA/ ITO with different blend ratio.

WORM memory mechanism in PEDOT: PSS or related composites has been a well-known effect^19^,^20^ while their memristor phenomena are still in debate or discussion^21^,^22^,^23^. To gain better insight on the governing mechanisms of the memristor mode, one needs to exclude spurious effect from metallic filaments mechanism from electromigration of top or bottom electrodes. While control experiments with PVA (Fig. 4a) can safely exclude filament mechanism, further validation is required. We performed further control experiments to elucidate or exclude the role of electrodes or electrodes’ filaments in the memristor mode of the blend. The polymer blends with weight ratio of PEDOT: PSS to PVA varied at 1: 5, 1: 10 and 1:30 were prepared. All the samples were fabricated at the same batch to avoid spurious effects of electrode fabrication. It can be seen from Fig. 5 that by adding more PVA (weight ratio 1:30), the current level will be reduced which can be attributed to the insulative properties of PVA. The current level dependence on the blend ratio further proves that the filament mechanism from electrode metal migration is not the governing mechanism.

**Figure 6:**
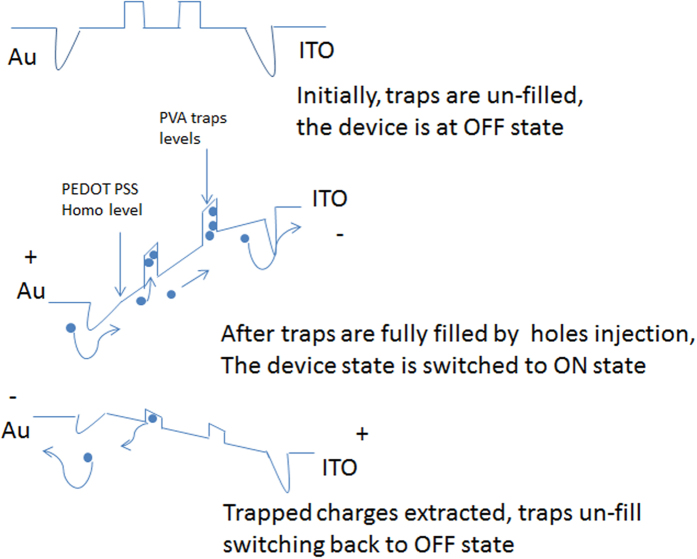
Charge trapping model to explain the memristor effect in PEDOT: PSS blend PVA system.

In memristor mode, the blend of PEDOT: PSS and PVA resembles the model system of traps induced resistive switching that was discussed extensively in literature. The charges injected from the contacts will be trapped by the conductive dopants in a similar manner to the doping of P3HT or PCBM into PI matrix or PMMA matrix^24^,^25^. This model has been successfully used to describe memory phenomena in various organic blends systems. Hence, we propose a mechanism to capture the essence of our observations by modifying the charge trapping model proposed by Lee and Kim^24^,^25^. Figure 6 presents the proposed modified charge trapping resistive switching model.

In the ITO bottom electrode device, traps can be filled by holes injection from anode and hence the device can be switched to ON state where charges can be trap-free transported and less scattered. In the proposed mechanism model, the traps are originated from PVA. The charge trapping characteristic of PVA has been observed many times in Organic Field Effect Transistor (OFET) and was due to dipoles from OH groups of PVA which can trap charges^26^. After being switched to ON state, by applying negative voltage on the Au top electrode, the trapped charges are extracted back and the device state will be reverted to OFF state or original state.

The device can also be fabricated on flexible ITO/ PET. Both WORM and memristor memory can also be obtained after bending the device for 20 times at 1.5 cm bend radius as shown in Fig. S2.

## Conclusion

In conclusion, the coexistence of WORM memory and memristor in the blend of PEDOT: PSS with PVA has been reported. At low voltage from −3 V to 4 V, the device exhibited memristor characteristic with self-crossing I–V behavior. Furthermore, the HRS (state 0) and LRS (state 1) could be cycled for more than 1000 times. After negative voltage of −8 V, the device’s resistance state was switched permanently to state _WORM_ which is smaller than both state 0 and state 1. The coexistence of these two resistive switching modes delivers potential for future reconfigurable multipurpose memory device with highly secured data storage options.

## Method

PVA (code name 363103 Alrich) and PEDOT: PSS (code name 483095 Alrich) were purchased from Sigma Aldrich and used without further purifying. The weight ratio of the PEDOT: PSS and PVA of 1 to 10 were prepared in DI water. Unlike the work of Huang and Ma where PEDOT: PSS was diluted by adding small amount of PVA^13^, we blended the PVA with PEDOT: PSS in higher weight ratio, so that charge trapping model could be applied in the same manner like doping Poly(methyl methacrylate) (PMMA) by Poly(3-hexylthiophene-2,5-diyl) (P3HT) or doping Polystyrene (PS) by Phenyl-c61-butyric acid methyl ester (PCBM) reported previously in literature^24^. The commercially available ITO coated glass electrode was cleaned by detergent and Acetone/ IPA mixture in ultrasonicator bath; the ITO was treated with oxygen plasma to induce hydrophilicity. The solution in 13 mg/ ml blend of PEDOT: PSS and PVA in DI water was spin coated at 1500 rpm to obtain nominally 75 nm thick films. The films were annealed at 120 °C in Ar filled glovebox for 2 h. The top gold electrodes were evaporated through shadow mask using thermal evaporator at 1 × 10^−4^ Torr and deposition rate at 0.005 nm/s using in- situ crystal quartz. Using gold electrodes (in 0.2 mm diameter), we avoid the complicated oxide interface between PEDOT: PSS and reactive metal electrodes such as Titanium or Aluminum^22^,^23^. Such oxide interface has been pointed out many times as the main factor for resistive switching in organic materials. Atomic Force Microscopy (AFM) was used for investigating surface morphology and conducting Atomic Force Microscopy (cAFM) was used to identify conducting domains and insulative domains. The AFM model used is P8 NT-MDT; we used diamond coated tips with radius of 50 nm and spring constant of 2–10 N/m for imaging electrical transport. Control film of PVA (75 nm) and PEDOT: PSS (90 nm) were made on cleaned ITO coated glass for comparison; devices were completed by gold evaporation as described above. Electrical bias was applied on top electrodes while bottom electrode was grounded. Sweep voltage was done at step 0.05 V. Film thickness was determined from surface profiler meter KLA TENCOR. The measurement was performed at 60% humidity and ambient condition unless separately noted. Circular electrode with size of 0.2 mm in diameter was used.

## Additional Information

**How to cite this article**: Nguyen, V. C. and Lee, P. S. Coexistence of write once read many memory and memristor in blend of poly(3,4-ethylenedioxythiophene): polystyrene sulfonate and Polyvinyl Alcohol. *Sci. Rep.* **6**, 38816; doi: 10.1038/srep38816 (2016).

**Publisher's note:** Springer Nature remains neutral with regard to jurisdictional claims in published maps and institutional affiliations.

## Supplementary Material

Supplementary Information
