# Supplementary material for: Coexistence of Write Once Read Many Memory and Memristor in blend of Poly(3,4-ethylenedioxythiophene): polystyrene sulfonate and Polyvinyl Alcohol
Source: Sci Rep. 2016 Dec 12;6:38816. doi: 10.1038/srep38816 (PMC5150231; doi:10.1038/srep38816)
Supplement: Supplementary Information [file srep38816-s1.pdf]

Supplementary information

Coexistence of Write Once Read Many Memory and Memristor in blend of Poly(3,4-ethylenedioxythiophene): polystyrene sulfonate and Polyvinyl Alcohol

Viet Cuong Nguyen and Pooi See Lee*

School of Materials Science and Engineering, Nanyang Technological University, 50 Nanyang Avenue, Singapore 639798, Singapore.

Email: pslee@ntu.edu.sg

Figure S1 shows I-V of the device testing in N_2 environment and O_2 environment. In N_2 testing, the chamber is first vacuumed to 1x 10^{-4} mbar then N_2 gas is purged into chamber. For O_2 testing, the chamber is open while flowing of gas stream is fed in proximity to device under test. There is no significant change in memristor pinched I-V of devices tested in those environments.

Figure S2 shows flexible device made from PEDOT: PSS blend PVA in ratio of 1-10 on ITO/ PET. Both memristor and WORM memory can still be obtained in the device under 20 times bending at radius of 1.5 cm.

Figure S3 shows cycling of device in memristor mode with different thicknesses such as 85 nm, 100nm and 10µm. Coexistence of WORM and memristor can be obtained at all the thicknesses. However, in thick film of about 10µm produced by drop casting solution of PEDOT: PSS blend PVA (PEDOT: PSS to PVA ratio 1-10 and concentration of 13mg/ml), memristor effect disappeared even only after third voltage sweep cycle as shown in Figure 3c below. Such disappearance effect stems from the complication of PEDOT: PSS and its composite with three dimensional3D phase separation [1, 2]. Furthermore, when thickness of the composite is increased to micron size, the amount of trapped water within inside the composite can be significant and it can deteriorate memristor performance even just after 3 voltage sweeps cycle

which is likely due to electrolysis of water and significant reactions of water with PSS^- to form PSS H [3].

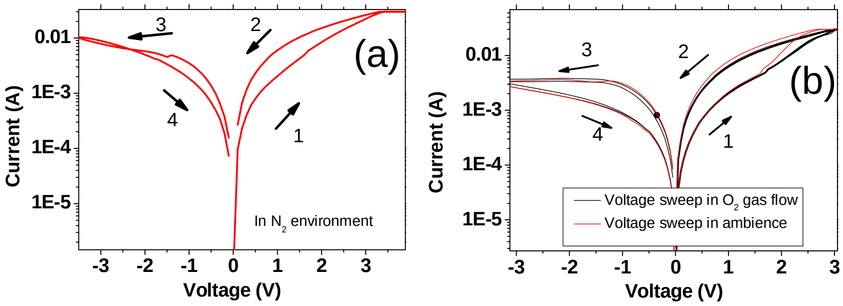

Figure S1| (a) Memristor I-V in N_2 environment (b) Memristor I-V in O_2 environment.

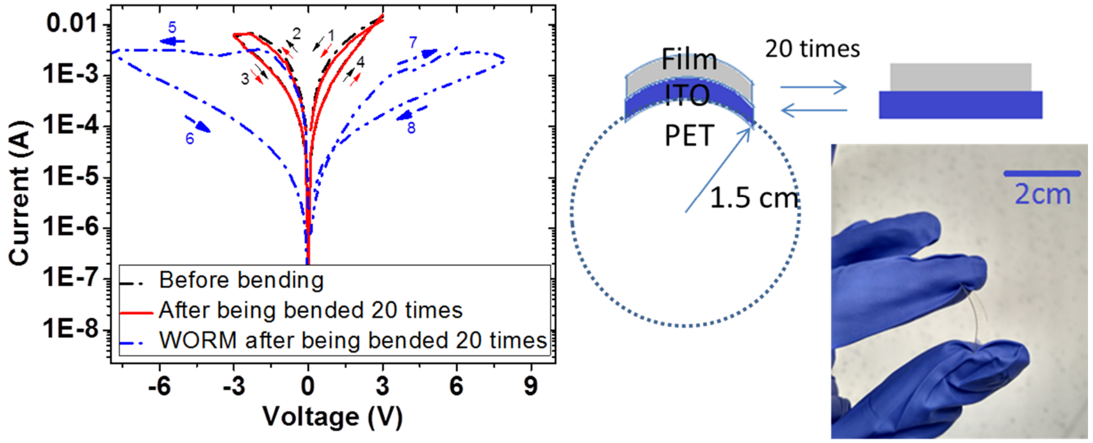

Figure S2| coexistence of WORM and memristor effect on flexible ITO/ PET. The color arrows are correspondent to color of I-V curves.

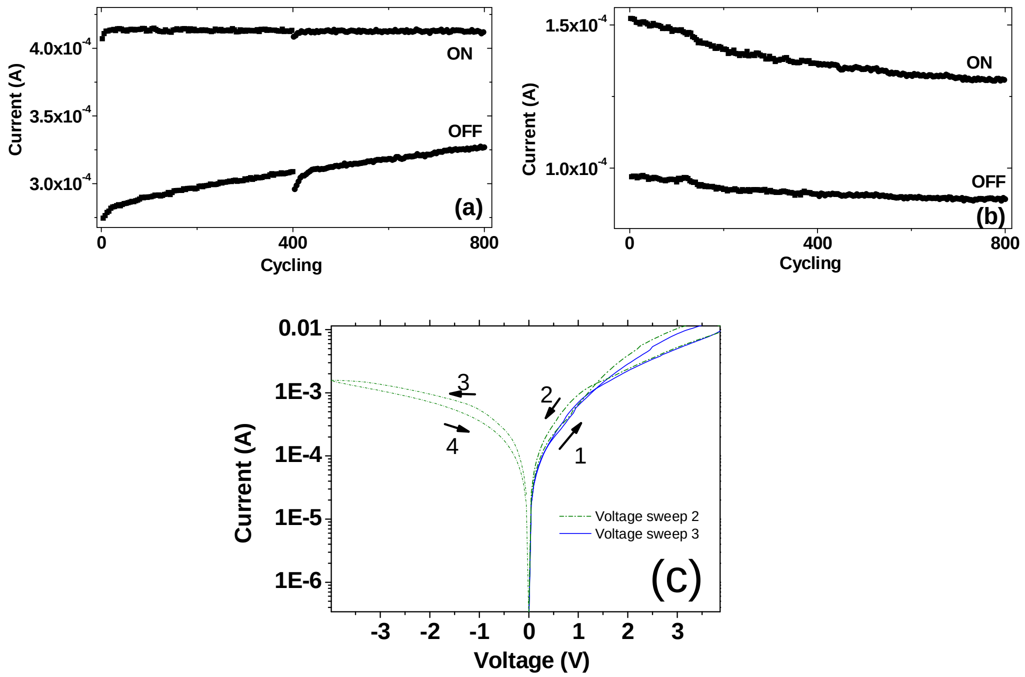

Figure S3| (a) cycling of device with thickness of 80 nm (b) cycling of device with thickness of 100 nm. (c) I-V sweep of device with thickness of about 10 μm. The device configuration is Au/ PEDOT: PSS blend PVA 1: 10/ ITO.

Reference

[1] L. Ouyang et al" Imaging the Phase Separation Between PEDOT and Polyelectrolytes During Processing of Highly Conductive PEDOT:PSS Films" ACS Appl. Mater. Interfaces, 7, 19764 (2015).

[2]M. Kemerink et al "Three-Dimensional Inhomogeneities in PEDOT:PSS Films" J. Phys. Chem. B, 10 , 18820 (2008).

[3]S. Moller et al "Electrochromic conductive polymer fuses for hybrid organic/inorganic semiconductor memories" J. Appl. Phys. 94, 7811 (2003).
